# Effect of SiO_2_ and MoS_2_ Particles as Lubricant Additives on Lubrication Performance in Sheet Metal Forming

**DOI:** 10.3390/ma18194605

**Published:** 2025-10-04

**Authors:** Krzysztof Szwajka, Tomasz Trzepieciński, Marek Szewczyk, Joanna Zielińska-Szwajka

**Affiliations:** 1 Department of Integrated Design and Tribology Systems, Faculty of Mechanics and Technology, Rzeszów University of Technology, ul. Kwiatkowskiego 4, 37-450 Stalowa Wola, Poland; 2 Department of Manufacturing Processes and Production Engineering, Faculty of Mechanical Engineering and Aeronautics, Rzeszów University of Technology, al. Powstańców Warszawy 8, 35-959 Rzeszów, Poland; tomtrz@prz.edu.pl; 3 Department of Component Manufacturing and Production Organization, Faculty of Mechanics and Technology, Rzeszów University of Technology, ul. Kwiatkowskiego 4, 37-450 Stalowa Wola, Poland; j.zielinska@prz.edu.pl

**Keywords:** electron beam melting, friction, ion implantation, mechanical properties, metal forming, steel sheets

## Abstract

Modifying lubricants with hard material particles improves lubricant performance by allowing the particles to penetrate the contact area and separate the contacting surfaces. The use of solid particles as additives in fluid lubricants presents a promising avenue for providing effective lubrication under high loads in sheet metal forming. This article presents the results of friction tests using the bending under tension friction tribotester. Low-carbon DC01 steel sheets were used as the test material. The main goal of the study was to determine the effect of lubricant modification by adding MoS_2_ and SiO_2_ particles and the modification of 145Cr6 steel countersamples on the coefficient of friction (CoF), changes in friction-induced surface roughness and friction mechanisms. The surfaces of the countersamples were modified using electron beam melting and the ion implantation of lead (IPb). It was found that increasing the SiO_2_ and MoS_2_ content in DC01/145Cr6 and DC01/IPb contacts under base oil lubrication conditions resulted in a decrease in the CoF value. For the countersample subjected to electron beam melting, considering all friction conditions, the CoF decreased between 31.9% and 37.5%.

## 1. Introduction

### 1.1. Friction in Sheet Metal Forming

Friction is one of the main limitations of sheet metal forming processes [1,2]. Friction causes excessive tool wear [3,4] and deterioration of the surface quality of drawpieces [5]. For decades, oils derived from fossil fuels were traditionally used in sheet metal forming processes. However, in the last two decades, research has been undertaken on the use of natural lubricants [6,7,8]. Numerous tribological tests have been developed to determine the coefficient of friction (CoF) in sheet metal forming processes: strip drawing tests, drawbead simulator tests and bending under tension (BUT) friction tests [9]. The BUT test considered in this paper has been the subject of scientific research and industrial testing in recent years. Andreasen et al. [10] proposed BUT equipment for testing the influence of the sliding speed and normal pressure on the limits of lubrication in DIN 1.3344 PM/DIN 17441 steel contacts. Normal pressure was found to have a significant effect on the limits of lubrication. Wiklund et al. [11] evaluated an advanced friction model for BUT testing which takes into account the properties of the lubricant, material and surface topography of the sheet metal. It was concluded that when the strain rates are increased, mixed lubrication is observed. Folle and Schaeffer [12] proposed an equation for the calculation of the CoF from the BUT test based on the radius of the pin and pin torque. It was found that the CoF value remained similarly close for the various equations. Trzepieciński and Lemu [13] investigated the effect of the surface roughness of the countersamples and lubrication condition on the CoF in deep drawing quality (DDQ)/145Cr6 steel contacts. The effectiveness of the lubricant increased with increasing roughness of the countersamples. Oliveira et al. [14] investigated the effect of countersample’s surface and pin diameter on the CoF in En AW-1100 aluminium alloy/AISI 430 steel contacts. They found that CoF increased with a decrease in the pin radius.

### 1.2. Why Is It Good to Use Solid Lubricant Additives

Modifying lubricants with hard powder particles improves anti-friction and anti-wear properties through different mechanisms [15]. Hard and brittle particles offer enhanced performance through polishing and rolling effects, where the particles create a smoother surface and act as ball bearings between the moving parts [16]. Moreover, additional particles in oils provide high-load lubrication by forming protective tribofilms through its layered structure [17,18]. The most universal additives with a wide range of applications, include molybdenum disulphide (MoS_2_) and silicon dioxide (SiO_2_) [19,20,21]. The effect of several additive lubricants such as SiO_2_ and MoS_2_, on coefficient of friction has been widely researched [22,23]. Xie et al. [24] used SiO_2_ and MoS_2_ nanoparticles as lubricant additives for AZ31 magnesium alloy/steel contacts. It was found that the MoS_2_-modified lubricant showed better lubricating properties in terms of the lubrication film stability and the load carrying capacity. Xie et al. [25] investigated the synergistic effect of SiO_2_ and MoS_2_ nano-additives in EOT5# mineral oil. The high lubrication performance of the oil tested was explained by the micro-cooperation of different nanoparticles with disparate morphologies. Mosleh et al. [26] dispersed MoS_2_ in commercial sheet metal forming fluids and evaluated their lubrication performance in a 440C steel/titanium sliding pair by using laboratory tribotesting. Modified lubricant reduced wear by 55–65% compared to unmodified fluid. Trzepieciński et al. [27] analysed the effect of SiO_2_ additives in rapeseed oil and sunflower oil on friction in HCT600X + Z/145Cr6 steel contacts. It was found that the lubricant with 5 wt.% of SiO_2_ particles was more effective in reducing CoF than the vegetable oils with an additional 1 wt.% of SiO_2_. Pang et al. [28] investigated the lubrication properties of SiO_2_/vegetable oil lubricant for metal forming applications. The modified lubricant exhibited better tribological performance than of base oil.

### 1.3. Motivation and Structure of the Paper

Despite the many advantages of natural lubricants, the automotive industry, which is the largest beneficiary of sheet metal components, still uses traditional petroleum-based oils. Modifying these oils with hard material powders is believed to improve their lubrication performance. The use of hard material particles as additives in lubricants demonstrates the potential for providing effective lubrication under high loads in sheet metal forming. The main aim of this study was to determine the effect of lubricant modification (by adding MoS_2_ and SiO_2_ particles) and modifying the surface of countersamples on the CoF and the friction-induced surface roughness sample changes in different contact pairs. Three types of 145Cr6 tool steel countersamples were used, differing in surface treatment methods. The surfaces of the countersamples were modified using electron beam melting (EBM), the ion implantation of lead, and combined EBM and ion implantation of lead. Friction test results for these samples were compared with the results for a countersample with an unmodified surface. To the best of the authors’ knowledge, similar BUT tests involving lubricants modified with powder particles and surface-modified countersamples manufactured by EBM, with the and ion implantation of lead, have not been performed previously. Section 2 of this paper presents the characteristics of the tested material and the test stand. The methods used to modify the countersample surface and the research plan are presented. Section 3 is divided into separate sub-sections devoted to the tribological analyses conducted. Section 3.1 presents the results and a discussion of the effect of countersample type and SiO_2_ and MoS_2_ particle content on the coefficient of friction. The effect of countersample type and lubrication conditions on the change in selected sheet metal surface roughness parameters is presented in Section 3.2. The main conclusions and interpretations are given in Section 4.

## 2. Materials and Methods

### 2.1. Test Material

The research involved the use of 0.8 mm thick low-carbon DC01 steel sheet (ArcelorMittal Poland S.A., Dąbrowa Górnicza, Poland). This steel material is widely used in many industries, such as the automotive (car) industry (where it is used to produce various body parts) and the manufacturing sector (where deep drawn housings are produced).

The widespread application of the test material is primarily related to its mechanical properties, which result from its chemical composition. The carbon content in this material reaches 0.06%, which increases its hardness and mechanical strength. The presence of manganese (up to 0.35%) improves plastic properties and facilitates sheet metal forming. Elements such as phosphorus and sulphur (in concentrations up to 0.025%) are important for corrosion resistance and weld quality during the welding process [29]. Low-carbon DC01 steel sheet has high formability at room temperature and, because of its high susceptibility to work-hardening, low-carbon sheets are formed under cold forming conditions. Because low-carbon steel is inherently soft and ductile, it can withstand large plastic deformations at room temperature without cracking, allowing the production of complex shapes for the automotive and other industries.

The basic mechanical properties of DC01 steel were determined in a uniaxial tensile test using a Zwick/Roell Z100 tensile testing machine (Zwick/Roell, Ulm, Germany) in accordance with the EN ISO 6892-1:2020 standard [30]. The surface topography of the DC01 steel sheet was measured in the as-received state and after friction testing. Surface roughness measurements were performed using a stationary T8000RC profilometer manufactured by Jenoptik AG (Jena, Germany).

### 2.2. Friction Testing Method

**Figure 1:**
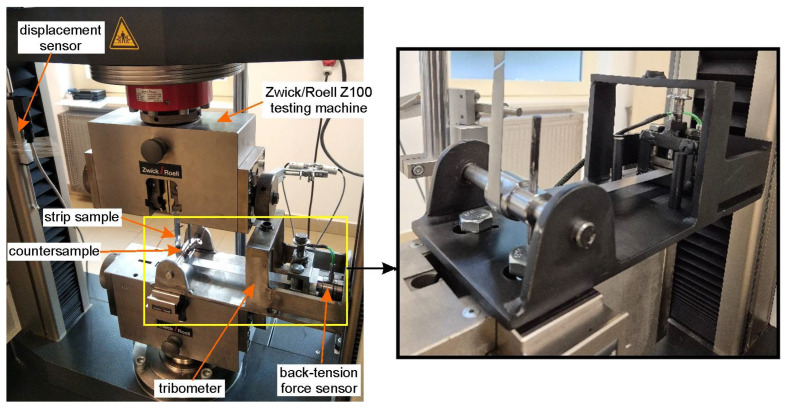
View of test stand.

The friction tests were performed using a self-designed bending under tension friction tester, as shown in Figure 1. The detailed design of this tribotester was previously described in [31]. The test samples were DC01 steel strips cut from a 0.8 mm thick sheet in the rolling direction. Each sample was 18 mm wide and 400 mm long.

The test involves stretching a strip of sheet metal around a 145Cr6 tool steel countersample with a diameter of 30 mm. The tests were conducted on the test stand shown in Figure 1, where one end of the sample was fixed in a specially constructed holder. The other end of the sample was mounted in the upper holder of a Zwick/Roell Z100 testing machine. The movement of this holder caused sample elongation whilst simultaneously bending it around the cylindrical countersample. The sample was stretched until it broke. The tests were carried out at an ambient temperature (21 °C). The testing machine was responsible for recording the front-tension force (FTF), while the back-tension force (BTF) was measured using a Kistler 9345B piezoelectric force sensor (Kistler, Winterthur, Switzerland). Based on both the FTF and BTH forces, the change in the friction coefficient μ value during the BUT test was determined according to Equation (1) [32]:

(1) $$\mu =\frac{2}{\pi }ln\frac{FTF}{BTF}$$

**Figure 2:**
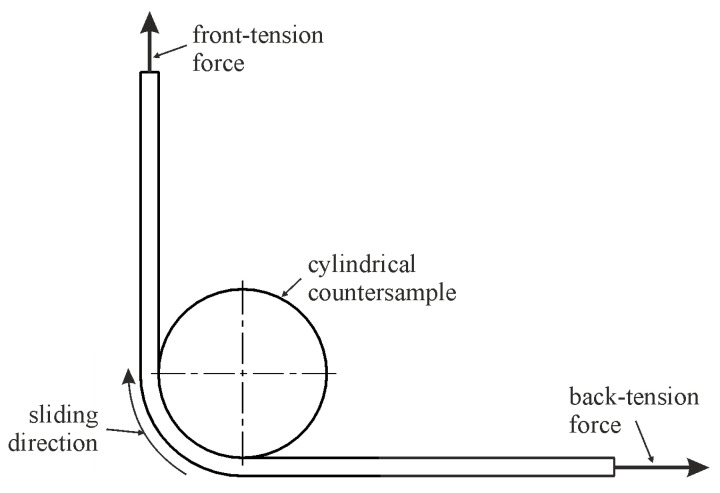
Schematic of bending under tension test.

The schematic of the BUT test shown in Figure 2 reflects the geometry of the rounded tool edges in sheet metal forming [9,11,13]. One of the main goals of the study was to determine the effect of modifying the countersample surface on the coefficient of friction and changes in strip sample topography during the BUT test.

As part of the investigations, three different variants of countersample surface modification were considered. The friction test results obtained for the modified countersamples were compared with the results of the tests conducted using unmodified (UM) countersamples. The countersample surface modification was performed using three methods: electron beam melting (sample denotation—EBM), ion implantation of lead (sample denotation—IPb) and combined ion implantation of Pb and EBM (sample denotation—IPb + EBM). A detailed description of the countersample surface modification procedures was already provided in a recent article [33].

During the tests, the effect of different lubrication conditions on the friction process was analysed. S100 Plus oil (Naftochem sp. z o.o., Kraków, Poland), characterised by a kinematic viscosity of 110 mm^2^/s, was used in the BUT tests. This oil is specifically designed for use in deep drawing operations. Additionally, S100 Plus oil was modified by adding SiO_2_ and MoS_2_ powders in three different mass concentrations: 1%, 5%, and 10%.

In order to precisely determine the influence of individual parameters on the material behaviour during the BUT test, a series of tests was carried out according to the research plan presented in Table 1.

## 3. Results and Discussion

### 3.1. Characteristics of Test Materials

The average values of the basic mechanical parameters of DC01 sheet metal are presented in Table 2. The parameter values are supplemented with standard deviations.

**Figure 3:**
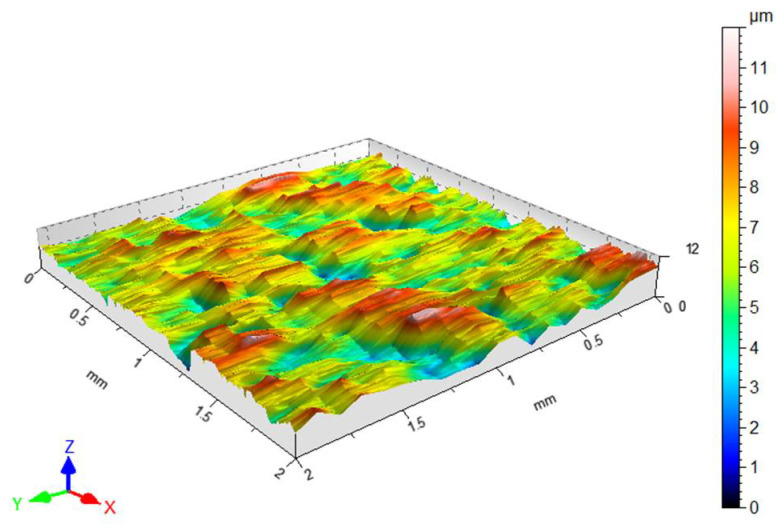
Surface topography view of the DC01 sheet metal in the as-received state.

The average surface roughness Sa for the as-received sheet metal is Sa = 1.25 μm and a view of the sheet’s surface topography is shown in Figure 3.

**Figure 4:**
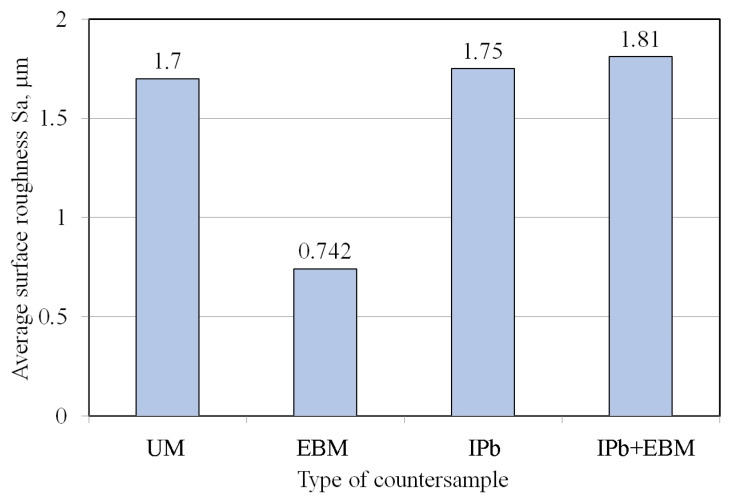
Average surface roughness of countersamples.

Values of basic surface roughness parameters of the countersamples are presented in Figure 4. The surfaces of the countersamples were characterised by the average surface roughness Sa [34]. The average roughness values varied between 0.742 μm and 1.810 μm depending on the type of countersample used.

**Figure 5:**
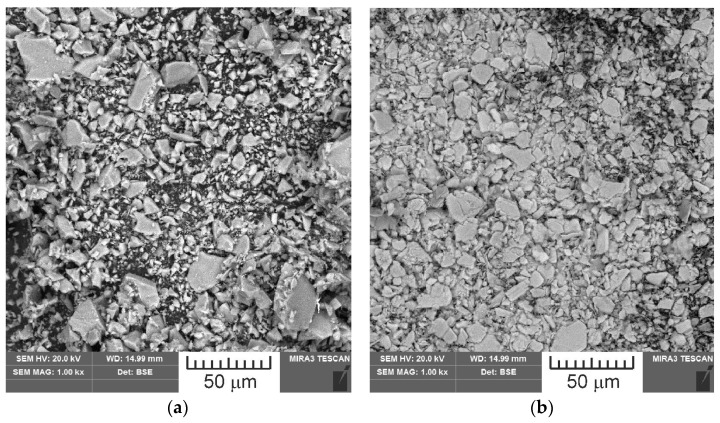
SEM images of powder materials: (**a**) SiO_2_, (**b**) MoS_2_.

**Figure 6:**
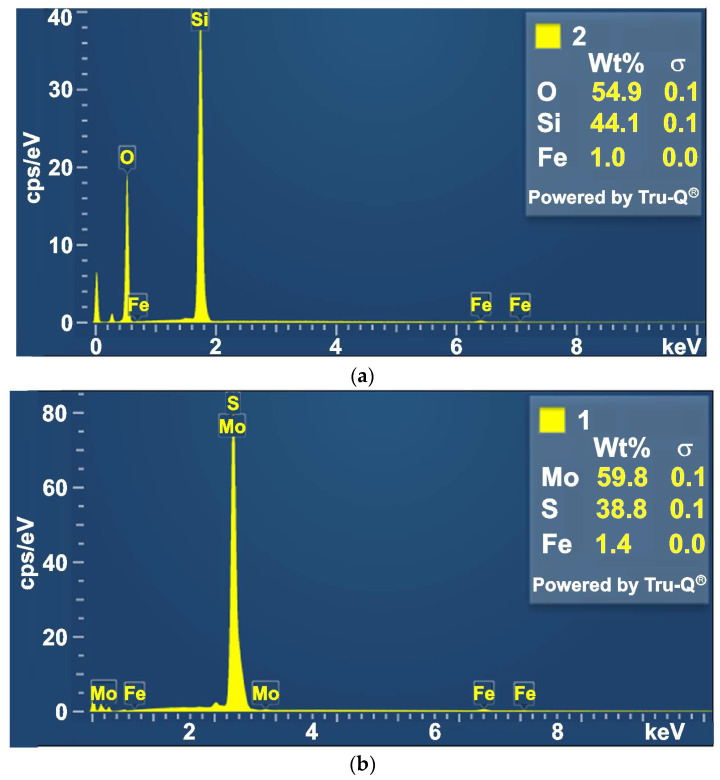
The content of elements in the powder: (**a**) SiO_2_, (**b**) MoS_2_.

The powders used in the study were examined for particle size and chemical composition. A TESCAN MIRA3 scanning electron microscope (SEM) was used for the microstructural analysis. Figure 5 shows SEM images of the powders used. The size of the major fraction of SiO_2_ particles was between 5 and 18 μm. The size of the major fraction of MoS_2_ particles was between 5 and 15 μm; however, smaller and larger particles were also observed. The results of the analysis of the powder’s chemical composition are presented in Figure 6.

### 3.2. Coefficient of Friction

**Figure 7:**
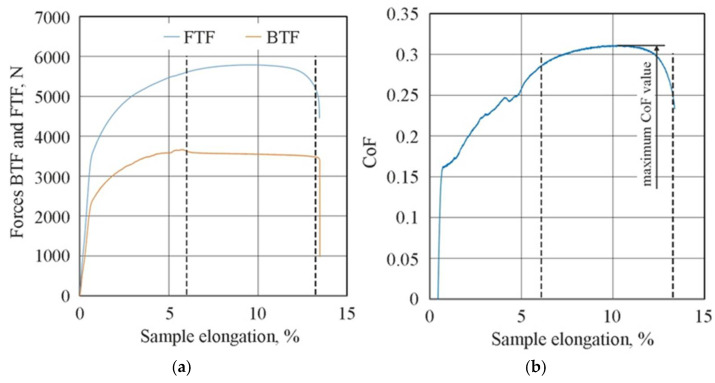
Changes in (**a**) force parameters and (**b**) CoF during BUT test under friction conditions with EBM countersample (dotted lines indicate the range of determining the maximum CoF value).

**Figure 8:**
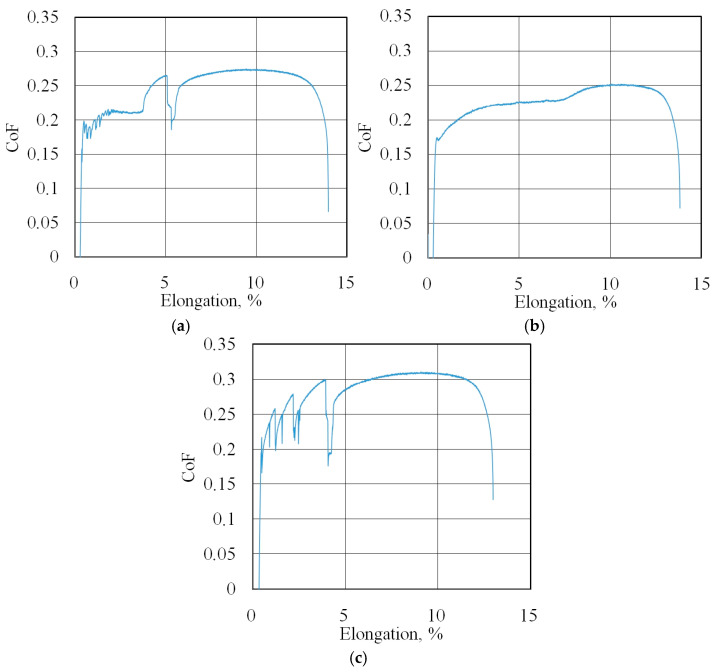
Changes in CoF (blue lines) during BUT test under dry friction conditions with the following countersamples: (**a**) UM, (**b**) IPb + EBM and (**c**) IPb.

Figure 7 shows the changes in the FTF and BTF during the BUT test and the corresponding change in the CoF value for the EBM countersample. Due to the continuous change in surface topography associated with sample stretching and the change in the sheet’s mechanical properties resulting from the work-hardening phenomenon, the CoF value is subject to continuous change [33]. Therefore, for comparison purposes, the maximum value of the coefficient of friction determined for sample elongation greater than 6% was adopted (Figure 8). Initially, the value of the force parameters increases rapidly as a result of overcoming the material’s resistance to deformation in the elastic strain range. Subsequently, the sample undergoes plastic deformations associated with the strain hardening. The strength of the strip sample is limited and at a certain point, there is a localised reduction in a specimen’s cross-sectional area, which corresponds to the maximum FTF; a subsequent reduction in the force parameters was observed.

**Figure 9:**
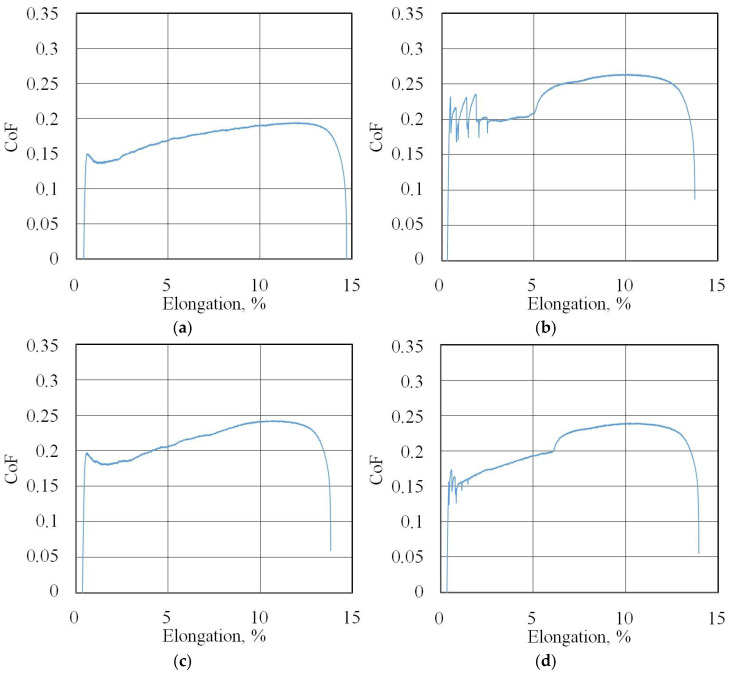
Changes in CoF (blue lines) during BUT test under lubrication with base S100 Plus oil with the following countersamples: (**a**) EBM, (**b**) UM, (**c**) IPb + EBM and (**d**) IPb.

**Figure 10:**
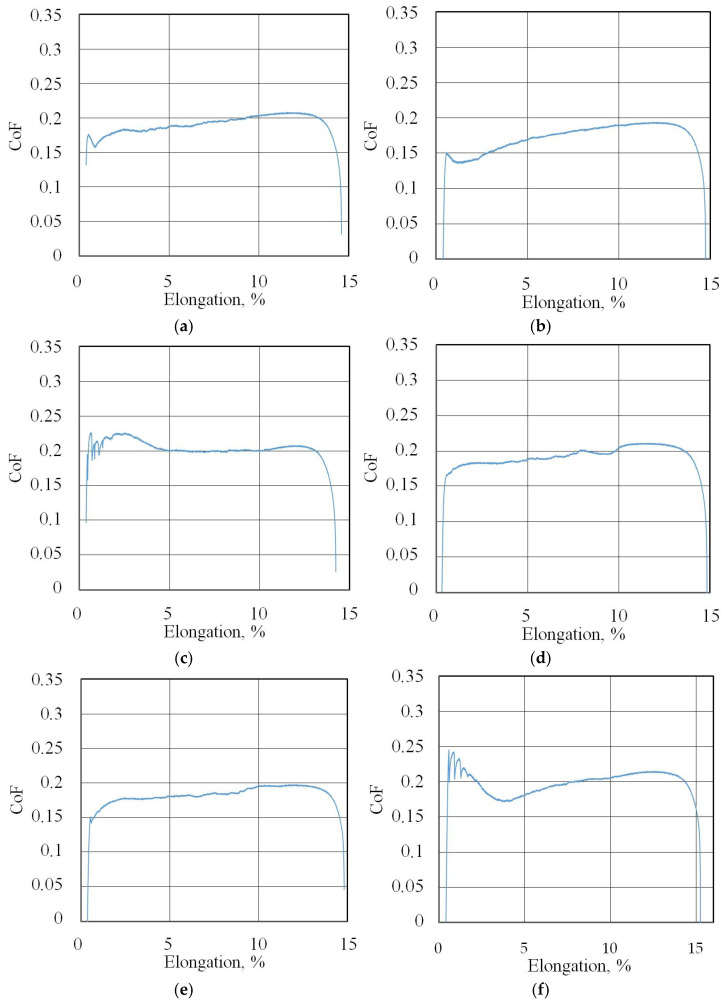
Changes in CoF (blue lines) during BUT test (EBM countersample) under lubrication with S100 Plus oil modified with (**a**) 1% SiO_2_, (**b**) 5% SiO_2_, (**c**) 10% SiO_2_, (**d**) 1% MoS_2_, (**e**) 5% MoS_2_, and (**f**) 10% MoS_2_.

**Figure 11:**
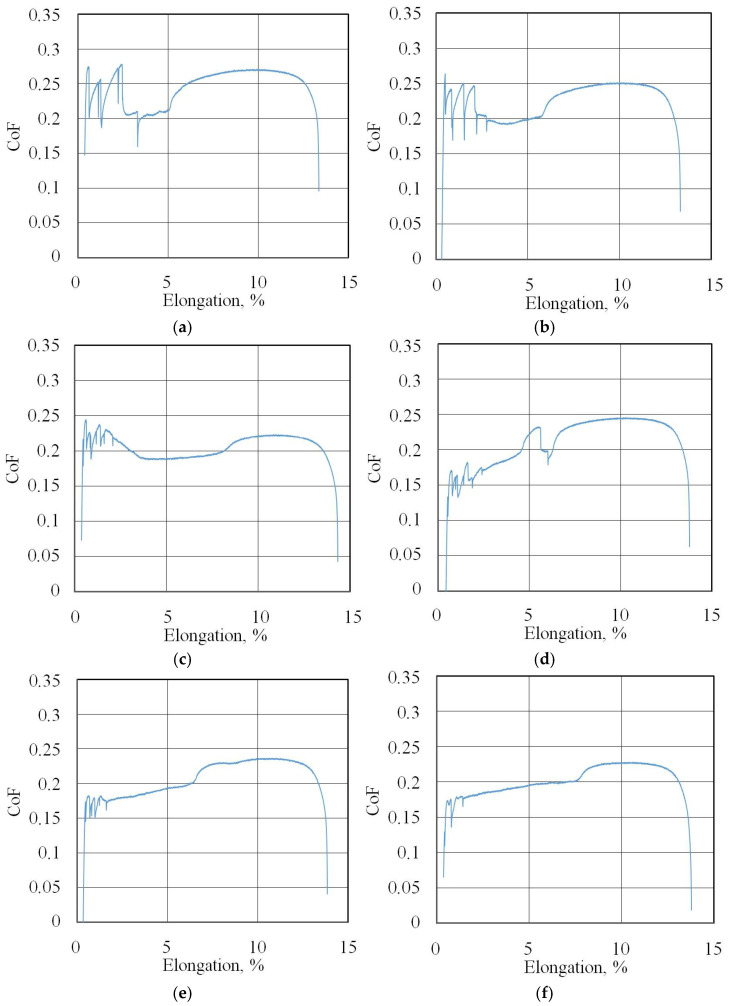
Changes in CoF (blue lines) during BUT test (UM countersample) under lubrication with S100 Plus oil modified with (**a**) 1% SiO_2_, (**b**) 5% SiO_2_, (**c**) 10% SiO_2_, (**d**) 1% MoS_2_, (**e**) 5% MoS_2_, and (**f**) 10% MoS_2_.

**Figure 12:**
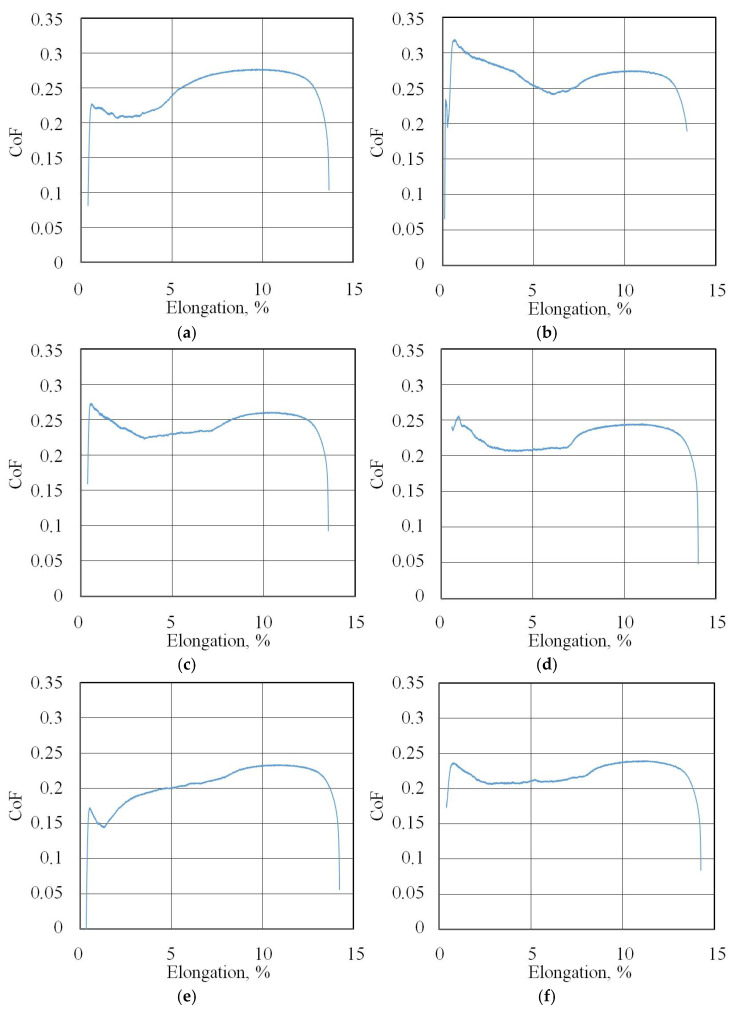
Changes in CoF (blue lines) during BUT test (IPb + EBM countersample) under lubrication with S100 Plus oil modified with (**a**) 1% SiO_2_, (**b**) 5% SiO_2_, (**c**) 10% SiO_2_, (**d**) 1% MoS_2_, (**e**) 5% MoS_2_, and (**f**) 10% MoS_2_.

**Figure 13:**
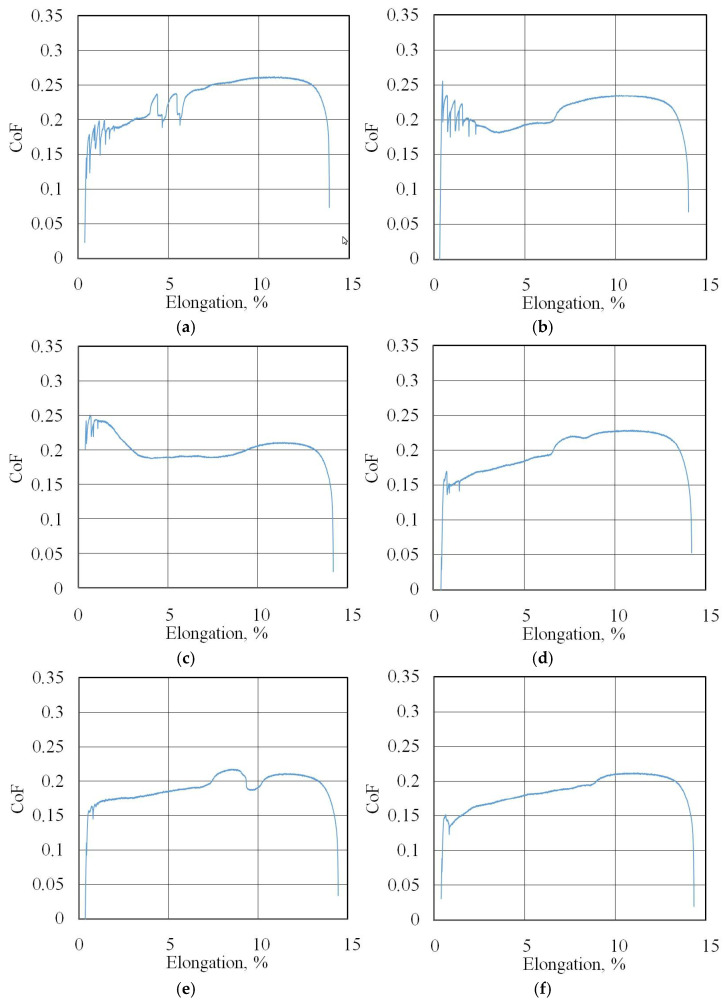
Changes in CoF (blue lines) during BUT test (IPb countersample) under lubrication with S100 Plus oil modified with (**a**) 1% SiO_2_, (**b**) 5% SiO_2_, (**c**) 10% SiO_2_, (**d**) 1% MoS_2_, (**e**) 5% MoS_2_, and (**f**) 10% MoS_2_.

The friction coefficient evolution curves (friction history) for all of the test conditions are presented in Figure 7b, Figure 8, Figure 9, Figure 10, Figure 11, Figure 12 and Figure 13.

**Figure 14:**
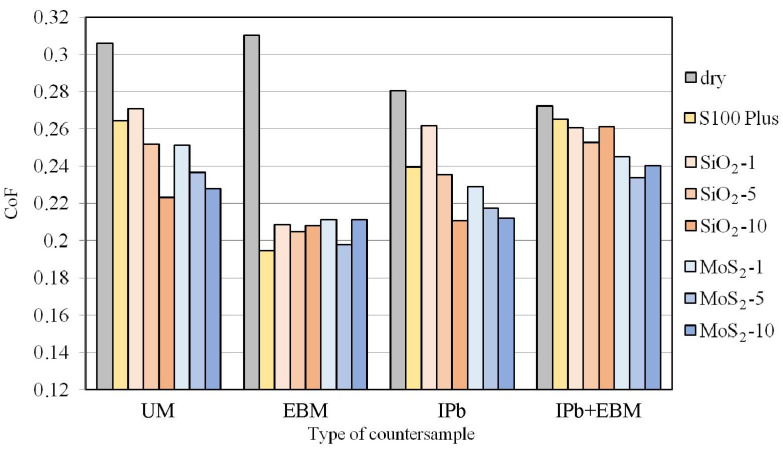
The influence of BUT test conditions on CoF.

A comparison of the maximum CoF values for all friction conditions specified in Table 1 is presented in Figure 14. In general, the expected relationship was observed, i.e., lubrication reduced the CoF value compared to dry friction conditions. Dry friction with UM and EBM countersamples provided a similar CoF value of approximately 0.31. The use of lubricant dramatically changed the coefficient of friction value. In the case of the UM countersample, its value depended on the content of SiO_2_ and MoS_2_ additives in the oil, and ranging between 0.223 (SiO_2_-10) and 0.271 (SiO_2_-1); this corresponds to a CoF reduction between 11.41 and 27.10%. In the case of the electron beam melted countersample, all friction conditions provided a CoF reduction between 31.9% (MoS_2_-1 and MoS_2_-10) and 37.5% (S100 Plus lubrication). For the UM and IPb countersamples, increasing the MoS_2_ and SiO_2_ content in S100 Plus oil resulted in a decrease in the coefficient of friction. During friction with the EBM and IPb + EBM countersamples, the lowest friction coefficient values were determined; modified oils were observed for oils containing 5 wt.% additives. This may be explained by the different topography of the countersamples, which determines the share of oil pockets and the distribution of asperity summits in the countersample surfaces.

The combination of high surface roughness and a low CoF for the countersamples does not have to be a contradiction. In sheet metal forming, a hard tool contacts a relatively soft sheet metal. High tool roughness, with appropriate lubrication, can provide large oil spaces which are capable of accommodating lubricant. Furthermore, high tool roughness reduces the actual contact area, limiting the metallic interaction of the friction pairs’ peaks. However, when the surface roughness is higher, surface asperities are embedded with each other, which will hinder friction pairs from sliding easily [35].

**Figure 15:**
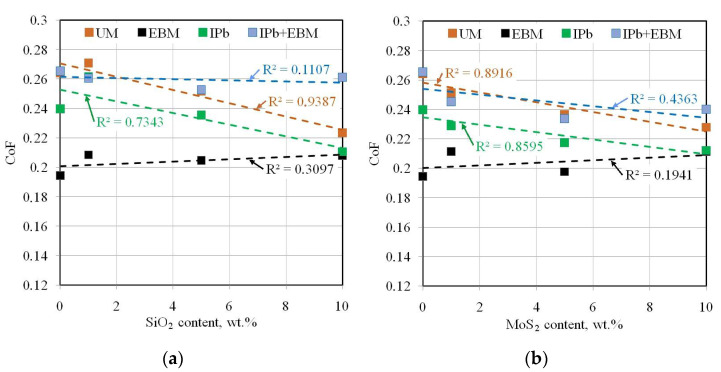
Effect of the addition of (**a**) SiO_2_ and (**b**) MoS_2_ to S100 Plus oil on the CoF.

The UM and IPb countersamples reduced the CoF with increasing MoS_2_ content in the lubricant (Figure 15). The largest reduction in CoF with the modified oil (by 19.5%) was observed for the IPb countersample and with oil containing SiO_2_ additive (Figure 15a). Only the EBM and IPb + EBM countersamples showed a similar effect on CoF for both powder additives. In the case of the EBM countersample, the SiO_2_ and MoS_2_ additives increased the friction coefficient compared to the lubrication conditions with pure S100 Plus oil. The opposite relationship was observed for the IPb + EBM countersample. The use of SiO_2_ (Figure 15a) and MoS_2_ (Figure 15b) additives reduced the CoF compared to the base oil.

In Figure 15, the COF of the UM and IPb countersamples decreases with the increase in the additional SiO_2_ and MoS_2_ content. For the EBM and IPb + EBM countersamples, the COF shows a trend of first decreasing and then increasing with the increase in the amount of SiO_2_ and MoS_2_, and reaches the minimum when the amount of SiO_2_ and MoS_2_ is 5 wt.%. These differences can be attributed to the higher peak–valley surface roughness Sz for the EBM (13.9 μm) and IPb + EBM (15.3 μm) countersamples compared to the remaining countersamples (10.2 μm for UM, 9.06 μm for IPb). According to the studies by Kong et al. [36] and Reeves et al. [37], lubrication effectiveness results from the interaction of particle size and surface roughness. High surface roughness in the countersamples causes faster lubricant film rupture due to the more concentrated interaction of surface roughness compared to surfaces with low asperities. Under these conditions, the number of particles has a decisive influence on the moment of lubricant film rupture. With a small number of particles, the effect of their addition to the base oil can be negligible. However, a large number of particles can increase friction by interfering with the surfaces and act as abrasive contaminants. As suggested by Xie et al. [25] and Zakani et al. [38], for a given surface roughness of the friction pair and contact pressures, there is an optimal concentration of particles for which a significant friction-reduction effect is obtained.

It is also important to note the effect of particle size on lubrication efficiency. For SiO_2_/MoS_2_ lubrication, smaller particle sizes are generally better, as they increase the surface area for more uniform lubricant film formation and better adhesion to contact surfaces, leading to reduced friction. However, extremely small nano-sized particles can agglomerate, hindering their ability to form a stable, continuous lubricating layer and negatively impacting performance. For the BUT test where deformation-induced surface roughness increases, larger particles are more effective in filling surface valleys as a result of the mending effect. Particles fill surface valleys and grooves, reducing surface roughness and diminishing the direct metal-to-metal contact.

### 3.3. Surface Roughness

The change in the topography of strip samples during the BUT test is related to sample elongation and the work-hardening phenomenon, which influences friction-induced contact mechanisms. Strain hardening influences the mechanical properties of the sheet metal: the sheet strength increases while the plastic properties decrease. Contact mechanisms primarily depend on the surface roughness of the contacting bodies but the sheet metal surface topography is subject to continuous evolution, while the tool surface topography remains constant or only changes to a small extent, ensuring stable conditions for the forming process of a series of products.

In order to analyse changes in the sheet metal topography, the basic parameters identified in the literature as being important in sheet metal forming were selected. Average roughness Sa is the most universal and basic parameter used in industry [39,40].

**Figure 16:**
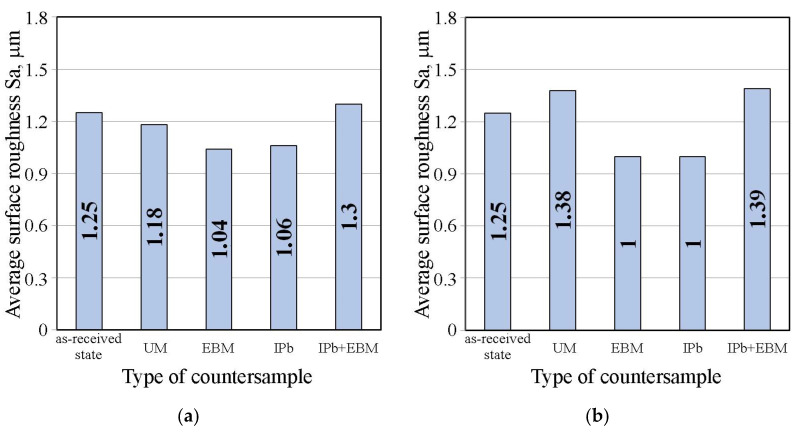
Influence of countersample surface treatment on basic surface roughness parameters of strip samples for (**a**) dry friction and (**b**) lubricated conditions with pure S100 Plus oil.

Figure 16a,b shows the effect of countersample surface treatment on the average surface roughness of strip samples for dry friction and lubricated conditions with pure S100 Plus oil, respectively. The average roughness Sa value under dry friction conditions (Figure 16a) and lubrication with oil without additives (Figure 16b) does not show significant changes induced by friction. The value of this parameter varies by approximately 11–20% depending on the friction conditions.

**Figure 17:**
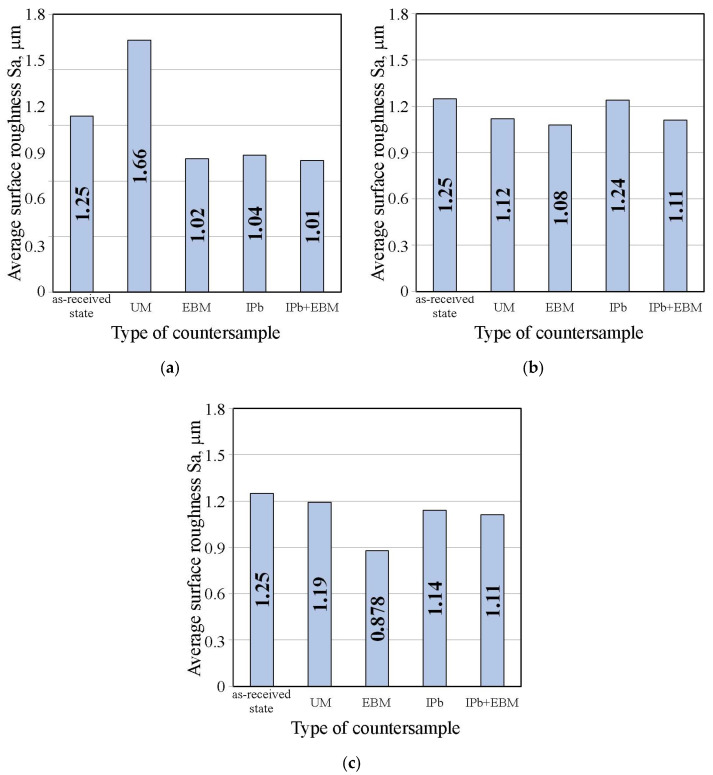
Influence of countersample surface treatment on basic surface roughness parameters of strip samples tested with S100 Plus oil with SiO_2_ added at (**a**) 1 wt.%, (**b**) 5 wt.%, and (**c**) 10 wt.%.

Figure 17 shows the effect of countersample surface treatment on average surface roughness of strip samples tested with S100 Plus oil with an SiO_2_ additive. In the case of oil with 1 wt.% SiO_2_, all countersamples with a modified surface provided a reduction in average surface roughness compared to the UM countersample. The largest increase in the Sa parameter under friction conditions with SiO_2_-modified oil was observed for the unmodified countersample (Sa = 1.66 μm). In friction conditions with oil containing 5 wt.% SiO_2_ (Figure 17b), a reduction in the Sa parameter value in EBM countersample was observed for all countersamples (up to 13.6%). Similarly, during friction with oil containing 10 wt.% SiO_2_, the friction process caused a reduction in average roughness between 4.8% (UM countersample) and 29.8% (EBM countersample).

**Figure 18:**
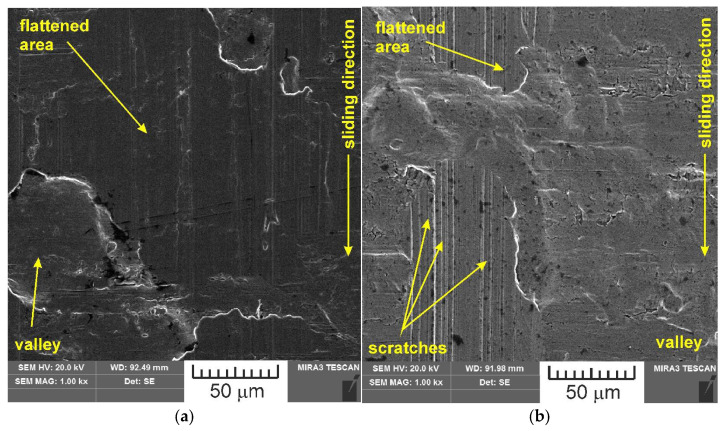

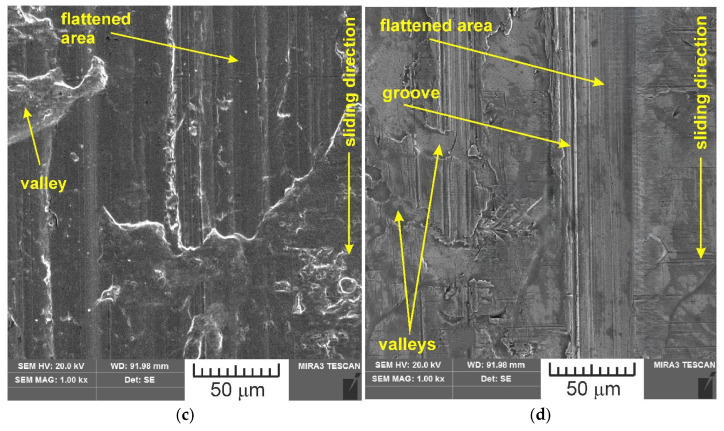
SEM images of the sample surface tested under dry friction conditions with the following countersamples: (**a**) EBM, (**b**) UM, (**c**) IPb + EBM and (**d**) IPb.

**Figure 19:**
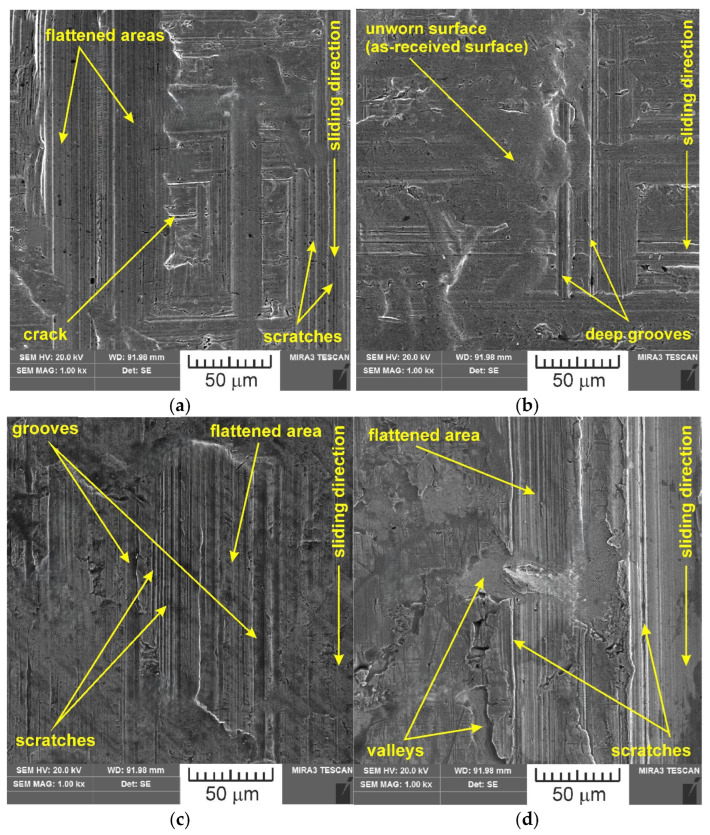
SEM images of the sample surface tested under lubrication with base S100 Plus oil with the following countersamples: (**a**) EBM, (**b**) UM, (**c**) IPb + EBM and (**d**) IPb.

**Figure 20:**
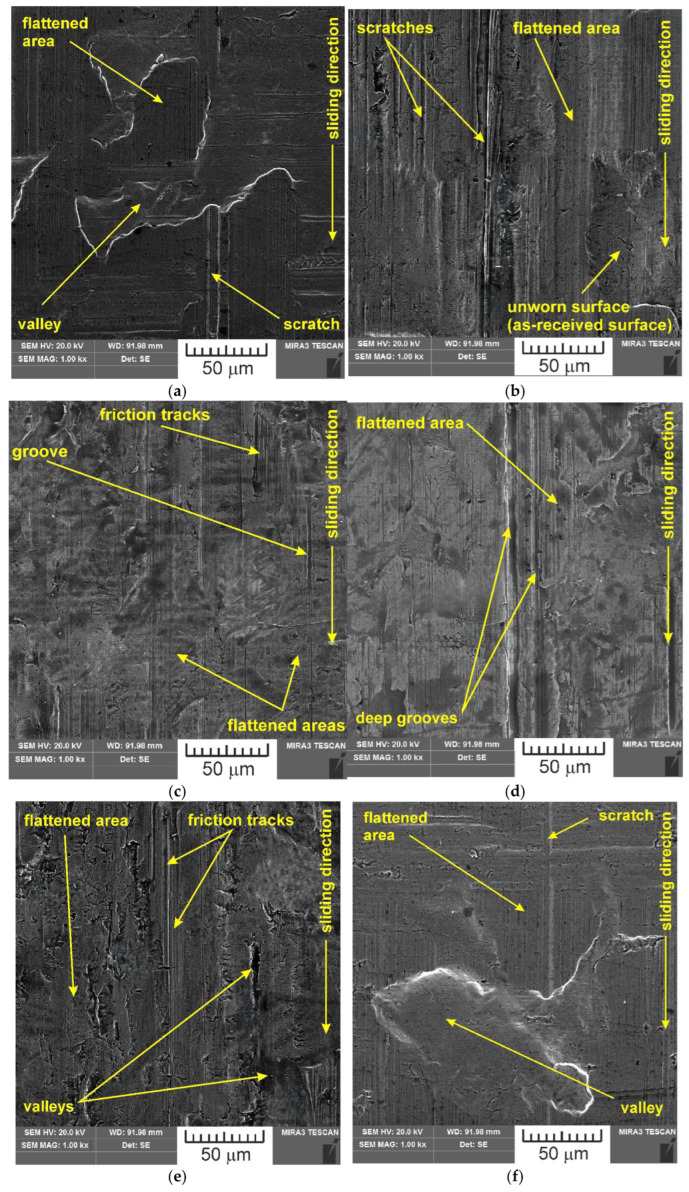
SEM images of the sample surface contacted with EBM countersample under lubrication with S100 Plus oil modified with (**a**) 1% SiO_2_, (**b**) 5% SiO_2_, (**c**) 10% SiO_2_, (**d**) 1% MoS_2_, (**e**) 5% MoS_2_ and (**f**) 10% MoS_2_.

**Figure 21:**
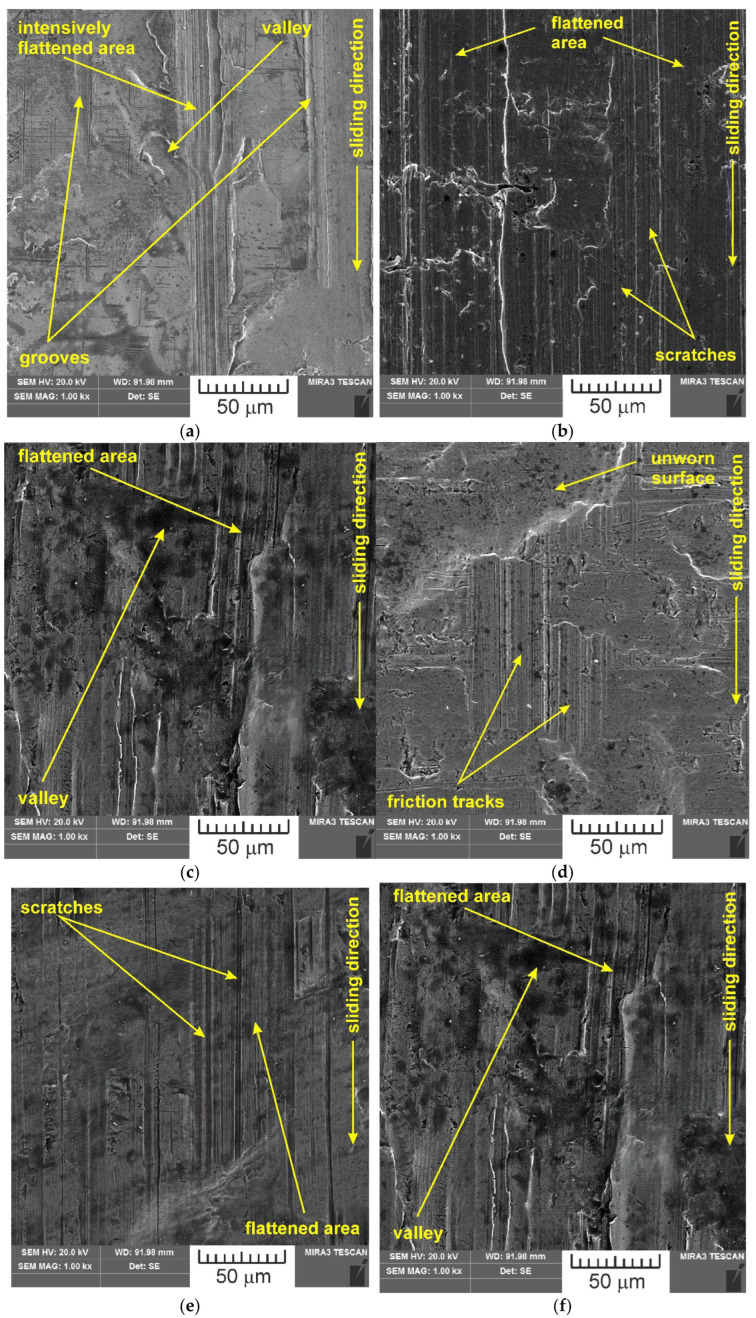
SEM images of the sample surface contacted with UM countersample under lubrication with S100 Plus oil modified with (**a**) 1% SiO_2_, (**b**) 5% SiO_2_, (**c**) 10% SiO_2_, (**d**) 1% MoS_2_, (**e**) 5% MoS_2_, and (**f**) 10% MoS_2_.

**Figure 22:**
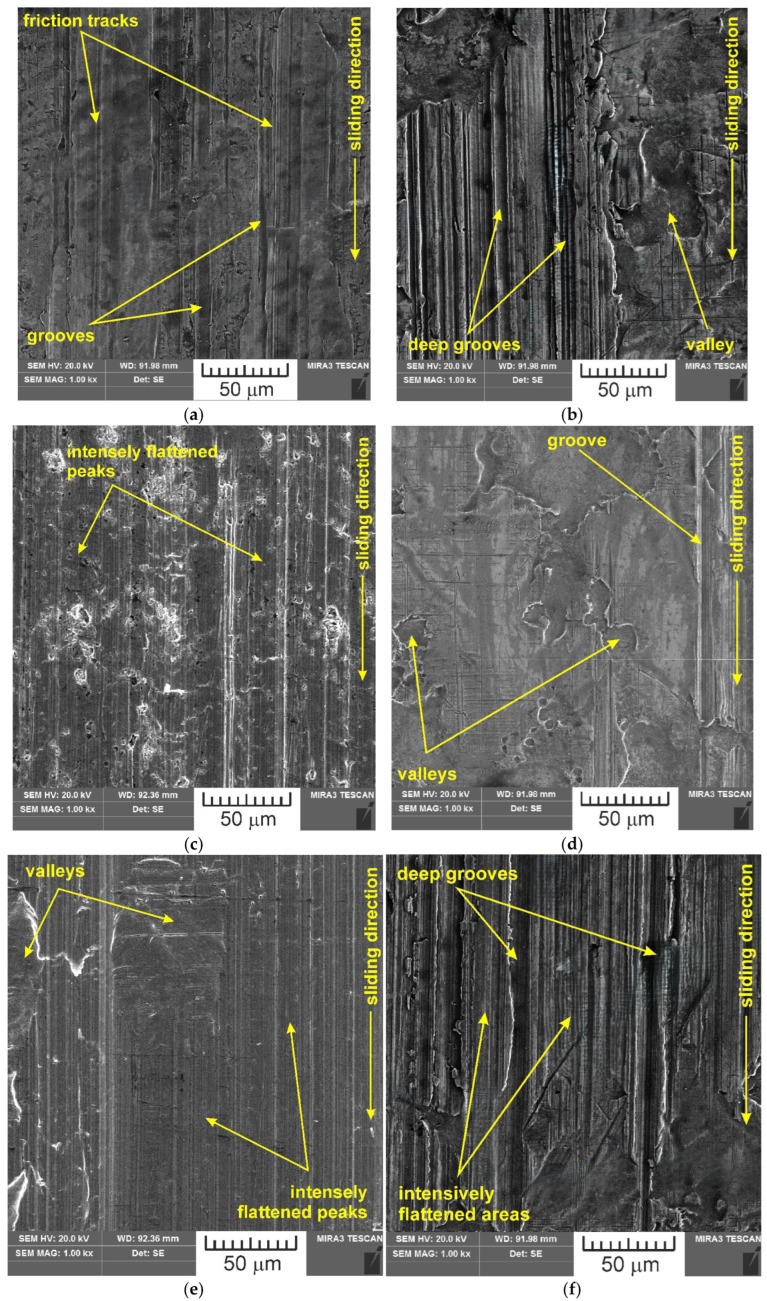
SEM images of the sample surface contacted with IPb + EBM countersample under lubrication with S100 Plus oil modified with (**a**) 1% SiO_2_, (**b**) 5% SiO_2_, (**c**) 10% SiO_2_, (**d**) 1% MoS_2_, (**e**) 5% MoS_2_, and (**f**) 10% MoS_2_.

**Figure 23:**
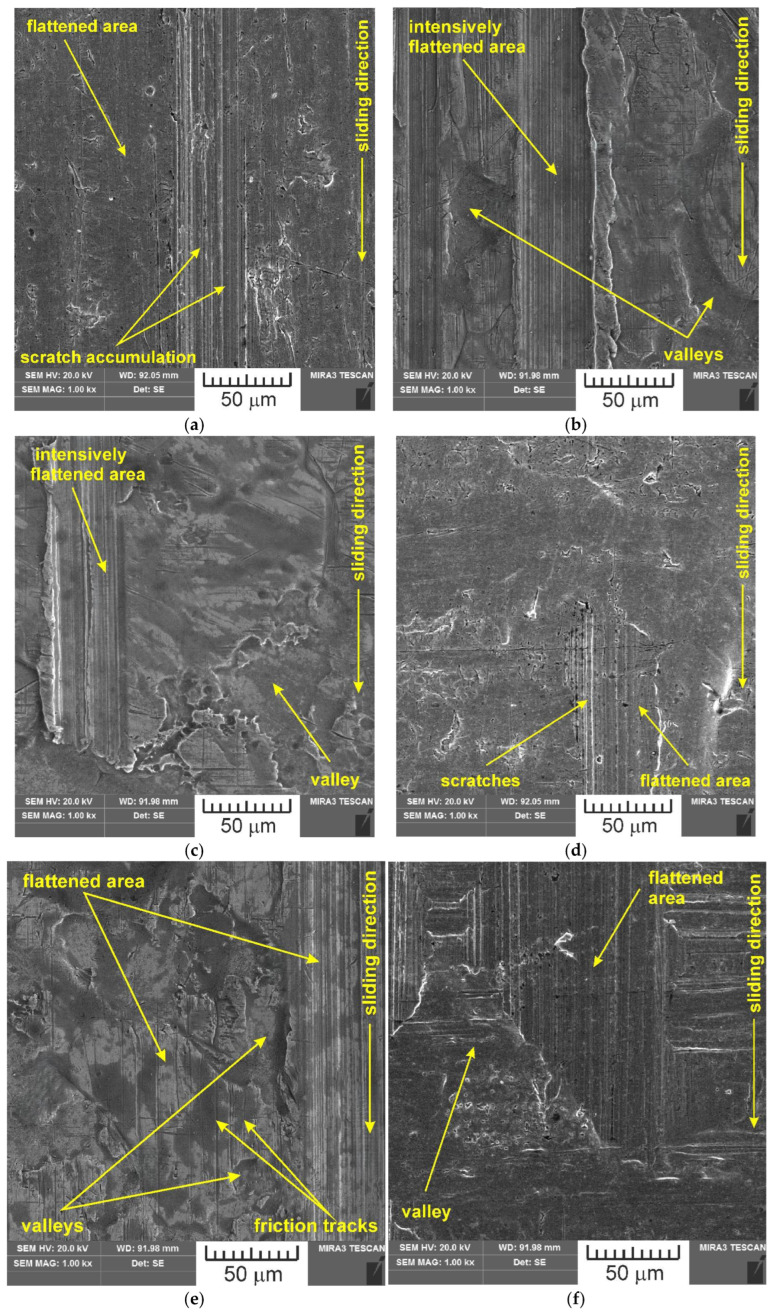
SEM images of the sample surface contacted with IPb countersample under lubrication with S100 Plus oil modified with (**a**) 1% SiO_2_, (**b**) 5% SiO_2_, (**c**) 10% SiO_2_, (**d**) 1% MoS_2_, (**e**) 5% MoS_2_, and (**f**) 10% MoS_2_.

SEM images of the sample which was surface tested under dry friction conditions with EBM, UM and IPb countersamples are presented in Figure 18. Figure 19 presents SEM images of the sample surface tested under lubrication with base S100 Plus oil with the EBM, UM and IPb countersamples. SEM images of sample surfaces tested under lubrication with oil containing SiO_2_ and MoS_2_ particles are presented in Figure 20, Figure 21, Figure 22 and Figure 23. In general, the sheet metal surface contains valleys created by the sheet metal manufacturing process and subject to deformation-induced evolution, as well as areas with flattened asperities. Scratches can be observed at the highest asperities, resulting from the interaction of tool asperities and sheet asperities. Deep grooves are the result of ploughing friction, which is defined as the contribution to friction force that occurs when a hard asperity in the countersample penetrates a softer sheet of metal.

**Figure 24:**
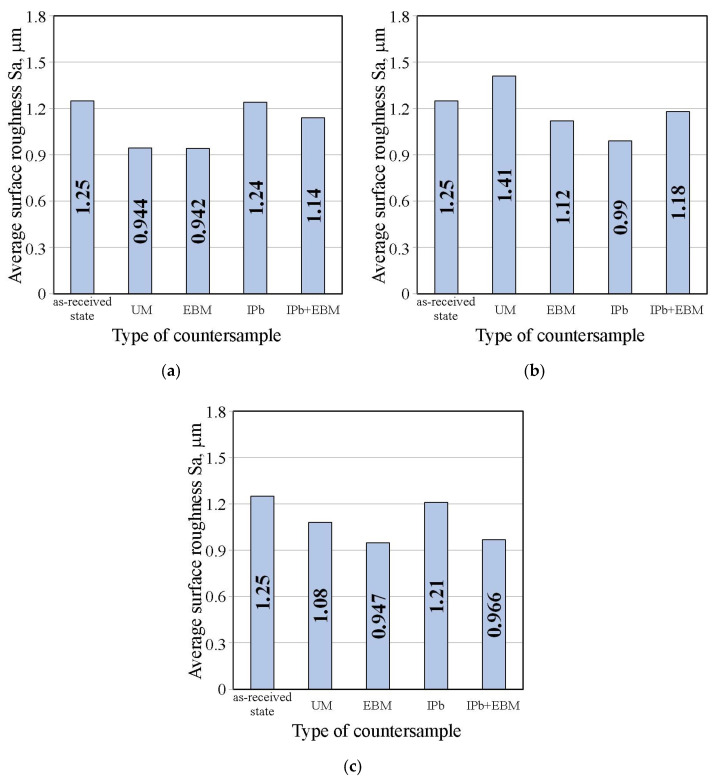
Influence of countersample surface treatment on basic surface roughness parameters of strip samples tested with S100 Plus oil with MoS_2_ added at (**a**) 1 wt.%, (**b**) 5 wt.% and (**c**) 10 wt.%.

The effect of surface treating the countersample on the average surface roughness of the strip samples tested with S100 Plus oil with the addition of MoS_2_, is shown in Figure 24. The average roughness Sa is the least sensitive to changes in surface topography after friction. During friction with oil containing 5 wt.% MoS_2_ (Figure 24a), the average roughness value varied between 0.8% (IPB) and 24.6% (EBM). During friction with oil containing 5 wt.% MoS_2_, the average roughness value varied between 12.8% (UM) and 20.8% (EBM). The change in average roughness observed for friction with the presence of oil containing 10 wt.% MoS_2_ (Figure 24c) was between 3.2% (IPb) and 24.2% (EBM).

**Figure 25:**
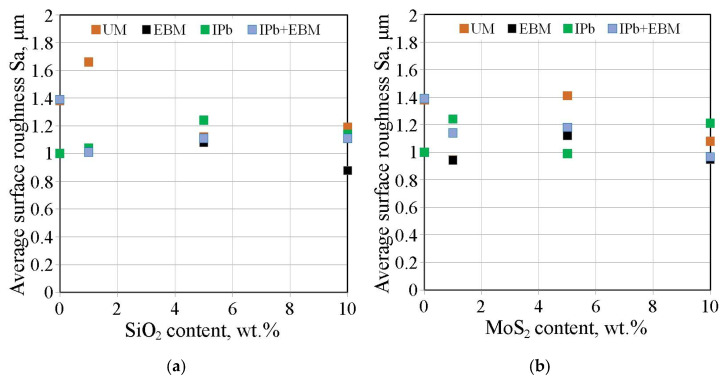
Influence of (**a**) SiO_2_ and (**b**) MoS_2_ content in S100 Plus oil on the average roughness Sa of strip samples after friction tests.

In the case of the IPb and EBM countersamples, the friction process resulted in a reduction in the average roughness value (Figure 25). Friction involving the unmodified countersamples was most sensitive to the SiO_2_ content (Figure 25a) and the addition of MoS_2_ (Figure 25b). In the absence of particles (or just a small amount: 1 wt.%), friction resulted in an increase in average roughness (Figure 25a). Under the conditions of lubrication with oil with the addition of 5–10 wt% SiO_2_ (Figure 25a), due to the frictional interaction of the strip sample with the countersamples, the average roughness Sa decreased, compared to the as-received surface.

**Figure 26:**
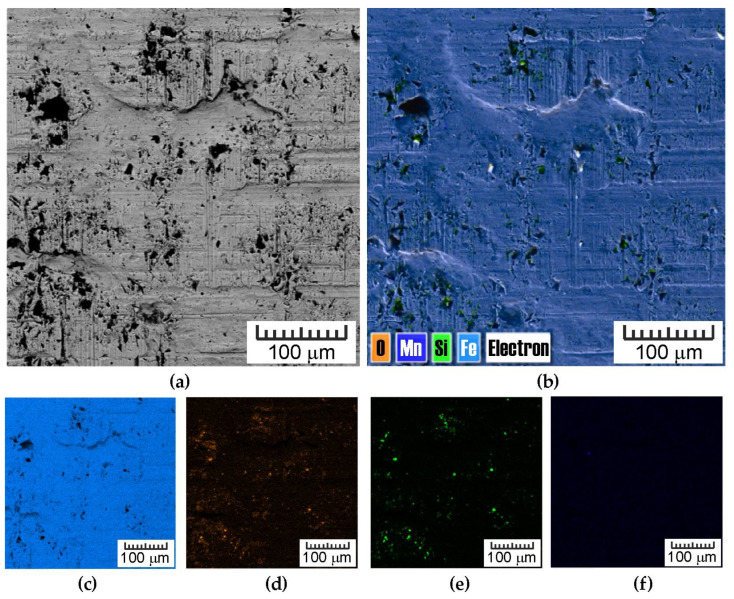
(**a**) SEM image (back-scattered electron detector mode) of strip sample after BUT test with oil containing SiO_2_ particles, (**b**) energy dispersive spectroscopy (EDS) layered image and EDS maps of the (**c**) Fe, (**d**) O, (**e**) Si and (**f**) Mn distribution.

Although interpreting the effect of friction conditions on topography changes is generally difficult, due to the synergistic interdependence of many factors, the indicated differences can be related to different particle properties. MoS_2_ exhibits a layered structure similar to graphite, while SiO_2_ exhibits a network structure. This makes MoS_2_ soft and cleavable, while SiO_2_ is hard (Mohs scale of 7 [41]) and its density is about half that of MoS_2_. Due to their high degree of hardness, SiO_2_ particles tend to embed themselves into the sheet surface (Figure 26).

## 4. Conclusions

The results of our research, presented in this paper, focused on determining the effect of lubricant modification (by adding MoS_2_ and SiO_2_ particles) and modifying the surface of countersamples on the CoF and changes in friction-induced surface roughness in the BUT friction test. The main research results are as follows:

- the expected relationship was observed, i.e., lubrication with pure and modified S100 Plus oil reduced the CoF value compared to dry friction conditions;
- in the case of the UM countersamples, the CoF value depended on the SiO_2_ and MoS_2_ additive content in the oil and ranged between 0.223 (SiO_2_-10) and 0.271 (SiO_2_-1);
- for the countersample modified by electron beam melting, considering all friction conditions, the CoF decreased between 31.9% (MoS_2_-1 and MoS_2_-10) and 37.5% (S100 Plus oil lubrication);
- increasing the SiO_2_ and MoS_2_ content when testing DC01/UM and DC01/IPb contacts under base oil lubrication conditions resulted in a decrease in the CoF value;
- in relation to the as-received surface, the value of the Sa parameter, in conditions of dry friction and lubrication with pure oil, varied in the range of 11–20%, depending on the friction conditions; for DC01/EBM and DC01/IPb contacts, the average roughness value decreased as a result of the friction process;
- SEM analysis of the countersamples surfaces identified adhesion, flattening, and ploughing as the main friction mechanisms.

## Figures and Tables

**Table 1:** Research plan.

Test No.	Countersample Type	Friction Conditions (Denotation)
1	EBM	dry
2		S100 Plus
3		S100 Plus + 1% SiO_2_ (SiO_2_-1)
4		S100 Plus + 5% SiO_2_ (SiO_2_-5)
5		S100 Plus + 10% SiO_2_ (SiO_2_-10)
6		S100 Plus + 1% MoS_2_ (MoS_2_-1)
7		S100 Plus + 5% MoS_2_ (MoS_2_-5)
8		S100 Plus + 10% MoS_2_ (MoS_2_-10)
9	UM	dry
10		S100 Plus
11		S100 Plus + 1% SiO_2_ (SiO_2_-1)
12		S100 Plus + 5% SiO_2_ (SiO_2_-5)
13		S100 Plus + 10% SiO_2_ (SiO_2_-10)
14		S100 Plus + 1% MoS_2_ (MoS_2_-1)
15		S100 Plus + 5% MoS_2_ (MoS_2_-5)
16		S100 Plus + 10% MoS_2_ (MoS_2_-10)
17	IPb + EBM	dry
18		S100 Plus
19		S100 Plus + 1% SiO_2_ (SiO_2_-1)
20		S100 Plus + 5% SiO_2_ (SiO_2_-5)
21		S100 Plus + 10% SiO_2_ (SiO_2_-10)
22		S100 Plus + 1% MoS_2_ (MoS_2_-1)
23		S100 Plus + 5% MoS_2_ (MoS_2_-5)
24		S100 Plus + 10% MoS_2_ (MoS_2_-10)
25	IPb	dry
26		S100 Plus
27		S100 Plus + 1% SiO_2_ (SiO_2_-1)
28		S100 Plus + 5% SiO_2_ (SiO_2_-5)
29		S100 Plus + 10% SiO_2_ (SiO_2_-10)
30		S100 Plus + 1% MoS_2_ (MoS_2_-1)
31		S100 Plus + 5% MoS_2_ (MoS_2_-5)
32		S100 Plus + 10% MoS_2_ (MoS_2_-10)

**Table 2:** Selected mechanical properties of DC01 steel.

Yield Stress, MPa	Ultimate Tensile Strength, MPa	Elongation, %
160.5 ± 2.76	284.5 ± 1.05	36.7 ± 0.23

## Data Availability

The original contributions presented in this study are included in the article. Further inquiries can be directed to the corresponding author.
